# N_2_O emissions from plants are reduced under photosynthetic activity

**DOI:** 10.1002/pei3.10015

**Published:** 2020-06-02

**Authors:** Klaus Schützenmeister, Katharina H. E. Meurer, Marco Gronwald, Antonia B. D. Hartmann, Dirk Gansert, Hermann F. Jungkunst

**Affiliations:** ^1^ iES Landau Institute for Environmental Sciences University Koblenz‐Landau Landau Germany; ^2^ Department of Soil & Environment Swedish University of Agricultural Sciences‐SLU Uppsala Sweden; ^3^ Osnabrueck University Osnabrück Germany; ^4^ Plant Ecology and Ecosystem Research Georg‐August University Göttingen Göttingen Germany

**Keywords:** N_2_O, photosynthesis, ash (*Fraxinus excelsior* L.), beech (*Fagus sylvatica* L.), rhizosphere, plant‐mediated

## Abstract

- New plant functions in the exchange of greenhouse gases between ecosystems and atmosphere have recently been discovered. We tested whether photosynthetic activity has an effect on N_2_O emission rates from incubated plant–soil systems.
- Two laboratory experiments were performed. One to unravel possible effect of photosynthetic activity on the net N_2_O ecosystem exchange for two species (beech and ash saplings). The other to account for possible effects from rhizosphere and aboveground plant parts separately (ash sapling only).
- Total N_2_O emissions from both plant and plant–soil systems were significantly lower under light than in darkness (31%–65%). The photosynthetic effect only applied to the aboveground plant parts.
- Underlying processes have now to be unraveled to improve our understanding of ecosystem functioning. This will improve modeling and budgeting of greenhouse gas exchanges between ecosystems and the atmosphere.

## 1 INTRODUCTION

Present research points to the importance of ecosystems to counteract human‐enforced climate change, because under sufficient nitrogen (N) availability plants are capable to partly fix carbon dioxide (CO_2_) emitted by human activity (Ainsworth & Long, 2005; Ainsworth & Rogers, 2007). Furthermore, interaction of plants and soils involved in GHG cycling are by‐passes of the oxic sink zone in soils, for example, methane (CH_4_) release to the atmosphere via aerenchyma (Li, Zhu, Bao, Wang, & Xu, 2016; Mosier, Mohanty, Bhadrachalam, & Chakravorti, 1990). This, however, mostly accounts for wetland plants, while most upland plants lack aerenchyma tissue. The effect of plants on N_2_O fluxes has received some attention in recent years and mainly shows how plants can consistently emit N_2_O even without aerenchyma (e.g., Chen, Boeckx, Shen, & Van Cleemput, 1999; Díaz‐Pinés et al., 2016; Goshima et al., 1999; Lenhart et al., 2015; Pihlatie, Ambus, Rinne, Pilegaard, & Vesala, 2005; Rochester, Wood, & Macdonald, 2015; Rueckauf, Augustin, Russow, & Merbach, 2004; Wen, Corre, Rachow, Chen, & Veldkamp, 2017; Zou, Huang, Sun, Zheng, & Wang, 2005). Moreover, there are some studies showing that plant leaves can actively emit N_2_O (Cheng, Sakai, Nishimura, Yagi, & Hasegawa, 2010; Hakata, Takahashi, Zumft, Sakamoto, & Morikawa, 2003; Lenhart et al., 2019; Machacova et al., 2016; Smart & Bloom, 2001). According to Zou et al. (2005), there are two mechanisms for the efflux of N_2_O from plants: N_2_O is either derived from the soil and transported by the plant or directly produced by the plant itself during N assimilation. So far, it is not always clear which of the mechanisms is dominant or whether they occur simultaneously under field conditions. Nevertheless, the transport of dissolved N_2_O through the plant happens via the transpiration stream (Chang, Janzen, Cho, & Nakonechny., 1998; Díaz‐Pinés et al., 2016; Pihlatie et al., 2005; Yu, Wang, & Chen, 1997) and the efflux can be from the plant stomata (Zou et al., 2005) and stem lenticels (Díaz‐Pinés et al., 2016; McBain, Warland, McBride, & Wagner‐Riddle, 2004). However, most laboratory studies in that research area solely focussed on the plants and ignored the contribution of soil. Nowadays, most measurements of N_2_O from plant–soil continua are derived from closed chambers with opaque walls (e.g., Jungkunst, Freibauer, Neufeldt, & Bareth, 2006; Kesik et al., 2005; Meurer et al., 2016). If the plants are small enough to fit into the chamber, then photosynthesis is not included as a potential driving force. Considering adult forest trees, measurements were done on the ground or the stem (Barba, Poyatos, & Vargas, 2019; Díaz‐Pinés et al., 2016; Machacova et al., 2016; Wen et al., 2017), inevitably excluding the forest canopy and therewith possible effects of photosynthesis on N_2_O fluxes. Additionally to their crucial role in buffering anthropogenic CO_2_ emissions, forests and forest soils have been shown to be of high importance for N_2_O exchange with the atmosphere (Butterbach‐Bahl & Kiese, 2005; Kesik et al., 2005; Reay, Dentener, Smith, Grace, & Feely, 2008; Stocker et al., 2013). However, an influence of photosynthesis was hardly even considered possible as the influence of abiotic factors such as soil temperature, bulk density, pH value, and soil moisture are much more evident. The exclusion of photosynthesis appears justifiable because photosynthetic carbon assimilation by leaves is remote from the process of N_2_O release by soil microorganisms to the atmosphere. Though Smart and Bloom (2001) showed that N_2_O can be emitted by wheat leaves during photosynthesis and Bruhn, Albert, Mikkelsen, and Ambus (2014) found that natural UV irradiation caused the ecosystem N_2_O emission to be ~30% higher than otherwise assumed using dark chambers as usual. Eddy covariance measurements always include the effect of photosynthesis but we are only aware of a few eddy covariance measurement that revealed diurnal N_2_O patterns of correlations to gross primary production (e.g., Zona et al., 2013). In general, a light‐dependent gas transport or N_2_O production mechanisms have been suggested in the literature (Jørgensen, Struwe, & Elberling, 2012), meaning that changes in illumination can directly affect N_2_O effluxes (Yu & Chen, 2009). In an extensive study including 32 plant species, Lenhart et al. (2019) found a strong relation between CO_2_ respiration and N_2_O effluxes, which was consistent over a broad range of changing environmental conditions (temperature and N supply). Though, they could not confirm the effect of light in their study.

Investigations of tree‐mediated N_2_O fluxes are rare and mostly restricted to seedlings and/or saplings under laboratory conditions (e.g., Machacova, Papen, Kreuzwieser, & Rennenberg, 2013; Pihlatie et al., 2005; Rusch & Rennenberg, 1998). So far, the highly likely influence of soil on plant‐derived N_2_O emission has hardly been considered and CO_2_ and N_2_O emissions from plants were measured under sterile conditions or using nutrient solutions to substitute the soil. Yet, the primary biogenic N_2_O sources are from soils (70%) and involve the microbial N transformations brought about by nitrification and denitrification (Mosier et al., 1998). Therefore, we intended to make the next step by measuring N_2_O emissions from trees in soil. This setup includes the competition for nutrients with microorganisms, explaining why net N_2_O emissions are lower from plant–soil systems than from soil alone. Despite the fact that N_2_O is obviously emitted by plants and it is therefore likely that photosynthetic activity will have some effect on the emissions, we hypothesized that photosynthetic activity will not have a relevant impact on net ecosystem N_2_O emissions. We expected the impacts to be small (<10%) and close or below detection limits. Therefore, field measurement campaigns could still neglect the photosynthesis effect.

## 2 METHODS

### 2.1 Net ecosystem experiment

#### 2.1.1 Plant and soil material

The soil used for the soil column experiment (stagnic Luvisol) was gathered from a mixed deciduous broad‐leaved forest in the Hainich National Park, Thuringia, Germany (51°04ʹN 10°30ʹE). The soil was sampled from the upper 10 cm (A_h_‐horizon) and homogenized by passing it through a 2 mm mesh sieve. Ash (*Fraxinus excelsior* L.) and beech (*Fagus sylvatica* L.) saplings (3‐ to 6‐years old) of approximately identical biomass (plant height 15–20 cm) were sampled in the same forest. The choice of the two tree species is based on the fact that they are the two most common broad‐leaved tree species in Central Europe and relatives to both species occur throughout the temperate zone. Moreover, ash and beech have differences in litter quality and water balance, which is why we expected them to behave differently throughout the experiment.

90 days after the beginning of the experiment, the soil was fertilized with 25 kg KNO_3_/ha. This fertilization was done to ensure sufficient N supply for N_2_O emissions, as fluxes decelerate in the mesocosms if not fertilized. Another KNO_3_ addition of 100 kg/ha was done 63 days later. The return of organic matter (mainly C) to the soil was simulated by applying 100 ml of a dissolved carbon (DOC) solution (5 g/L powdered ash litter). This C:N fertilization assured identical starting conditions between the treatments (C:N 11.8 with 1.82% organic carbon (C_org_)). To determine the concentrations of nitrate ( ${\text{NO}}_{3}^{-}$ ) and ammonium ( ${\text{NH}}_{4}^{+}$ ), soil samples were analyzed with the continuous flow injection colorimetry (SAN + Continuous Flow Analyzer, Skalar Instruments) at two dates within the experiment (Table 1). Nitrate was determined with the copper–cadmium‐reduction method (ISO 13395), and ${\text{NH}}_{4}^{+}$ with the Berthelot reaction method (ISO 11732).

**TABLE 1:** Soil properties of the soil used in the column experiment. ${\text{NO}}_{3}^{-}$ and ${\text{NH}}_{4}^{+}$ contents are given as means ± *SE*

Treatment	Sand	Silt	Clay	pH_KCl_	Prior to 1st fertilization		End of experiment	
					${\text{NO}}_{3}^{-}$	${\text{NH}}_{4}^{+}$	${\text{NO}}_{3}^{-}$	${\text{NH}}_{4}^{+}$
	g/kg				mg/L			
Ash (*Fraxinus excelsior*)	2.9	56.5	40.6	5.3	6.0 ± 1.4	0.1 ± 0.0	6.2 ± 0.0	0.2 ± 0.0
Beech (*Fagus sylvatica*)					6.3 ± 2.1	0.1 ± 0.0	9.4 ± 0.0	0.1 ± 0.0
Bare soil					11.3 ± 2.9	0.2 ± 0.0	11.2 ± 2.0	0.3 ± 0.1

### 2.2 Experimental setup

Fifteen acrylic glass cylinders (h = 50 cm; diameter, *d* = 17 cm) were filled with 5 kg of freshly sieved (2 mm) soil and planted with the saplings. The cylinders were transparent in order to enable photosynthetic active radiation (PAR) to pass. This resulted in the three treatments: ash, beech, and bare soil. The soil columns were placed randomly in a greenhouse with a steady air temperature of 20°C and a relative air humidity of 80%. Illumination (12 hr) was maintained by lamps (PAR: 203 ± 10 µmol m^2^ s^−1^ PPFD; Eye Lighting, Clean Ace). The water‐filled pore space (WFPS) of each column was adjusted to 75%–80% and controlled once a week. The setup allowed the determination of net CO_2_ uptake (plant assimilation) under illuminated conditions and dark CO_2_ emission (all related to the soil surface area). The measurements were performed under (a) PAR conditions and (b) under dark conditions using a black cloth. The order of light before dark measurements was changed every day to avoid a bias by the order of dark to light measurements. Measurements were performed biweekly at 8 a.m. The experiment ran for 183 days in total and N_2_O fluxes were low and close to the resolution of the gas chromatograph in all treatments. To ensure the accuracy of the data and be able to detect differences between the treatments, the dataset used in this study has been reduced to 14 samplings and a timespan of 58 days (35 days after the first fertilization; 25 kg KNO_3_/ha).

#### 2.2.1 Trace gas sampling

We collected headspace gas samples (30–32 cm above soil surface) at 0, 10, and 20 min, after chamber closing using 60 ml syringes. The gas concentrations were analysed with an auto‐sample, computer‐controlled (Probe 64+1, V1.31) gas chromatograph (Shimadzu GC‐14B). N_2_O was detected by a ^63^Ni electron capture detector. A linear regression was used to calculate the increase or decrease in gas concentrations, following Lessard, Rochette, Gregorich, Desjardins, and Pattey (1997). The influence of illumination was identified by the differences between N_2_O fluxes under light (PAR) and dark conditions, whereby negative Δ indicated a reduction of N_2_O fluxes under PAR conditions. The gross plant assimilation (Δ CO_2_) was calculated as Δ CO_2_ = CO_2_ light (photosynthesis)−CO_2_ dark (respiration). The relationship between Δ N_2_O and Δ CO_2_ was tested by linear regression after correcting the dataset for outliers by considering each data point below or above 1.5 times the interquartile range as being too far from the central values to be reasonable. This led to exclusion of a total of 5 data points for the ash and 3 data points for the beech treatment. To generalize our findings, in the further, Δ will be expressed as a percentage reduction, that is, as ((PAR‐dark)/dark)*100.

### 2.3 Day‐night test

To negate a possible diurnal trend in the effect of photosynthesis, we used the ash treatment (columns 11, 12, 14, 16, and 17) for a one‐time 24‐hr measurement campaign with samplings in 3‐hr intervals. WFPS was adjusted to 75%–80% and calculated for every gas sampling time, based on the column weights after the experiment and the assumption that WFPS decreased linearly during the experiment by evapotranspiration. As it had been shown that the sequence of the measurements did not have an impact on the N_2_O fluxes, samplings were first made under exclusion of photosynthesis and afterwards during photosynthesis. Gas samples were taken exactly as described above (see *Trace gas sampling*), that is, air was sampled via syringes and samples were analyzed in the gas chromatograph.

### 2.4 Plant flux experiment

To be able to negate that the observed reduction in N_2_O emissions was primarily soil‐driven, we measured emissions from above‐ and belowground parts of ash saplings separately in a shorter experiment (10 samplings in total with bi‐daily measurements). In this experiment, we only added a very small amount of mineral soil to mimic the rhizosphere. For that reason, the experimental period was kept to 10 days.

#### 2.4.1 Experimental setup

**FIGURE 1:**
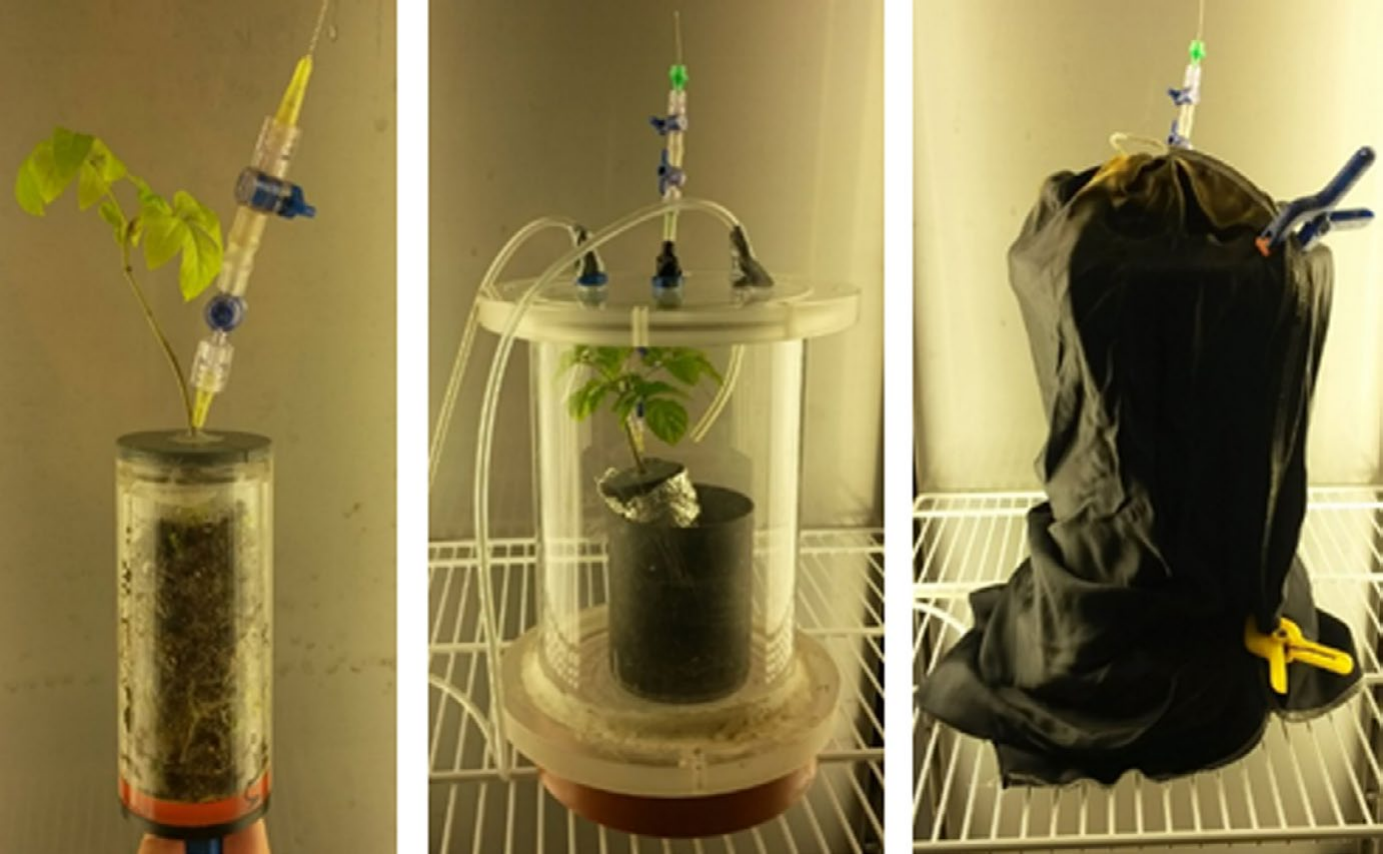
Setup for gas measurements from belowground (left) and aboveground plant parts under light (middle) and dark (right) conditions

Soil material was filled into six acrylic cylinders (*h* = 10 cm, *d* = 5 cm) and planted with ash saplings (height 8–14 cm). To separate the belowground and aboveground plant parts, an adjusted lid with a gap for the stem and a tube for belowground gas extraction was on top of the acrylic cylinder (Figure 1). The lid was sealed airtight to the cylinder. To avoid light penetration and, consequently, the growth of further biomass, for example, algae, in the space between soil and lid (3.0–3.6 cm), the acrylic cylinder was wrapped in aluminum foil. The ash saplings were placed randomly in a greenhouse with 20°C and 9.5 hr illumination per day. For gas measurements, the ash saplings were put into the columns used in the previous experiment (*h* = 50 cm; *d* = 17 cm; Figure 1) and gas samples for N_2_O were taken manually and using syringes and stored in 12.5 ml Labco vials until analysis at the gas chromatograph. A total number of three gas samples were taken over a period of 30 min (at 0, 15, and 30 min). Gas samplings were taken every other day and fluxes were calculated using linear regression (Lessard et al., 1997). The measurements started one day after all ash saplings had been fertilized with 200 kg N/ha as KNO_3_. Seven days after the fertilization, the ash saplings were irrigated until the soil became waterlogged. Measurements were done under (a) PAR conditions and (b) dark conditions. Cumulative fluxes were calculated by first linearly interpolating between the days and summarizing over the experimental period.

Correction for outliers, that is, data points below or above 1.5 times the interquartile range, led to the exclusion of 15 and 11 data points for the aboveground and rhizosphere treatment, respectively. Just like the net ecosystem experiment, ΔN_2_O is expressed as ((dark‐PAR)/dark)*100.

### 2.5 Statistical analyses

Statistical analyses were done in R (R Core Team, 2018) and data were processed using the *openxlsx* (Walker, 2019) and *plyr* (Wickham, 2011) packages. Figures were made with the ggplot (*ggplot2* package; Wickham, 2016) and plot_grid functions (*cowplot* package; Wilke, 2019).

The design of all experiments allowed for pairwise statistics because identical tree–soil systems (*n* = 5) were measured in darkness and thereafter again under light conditions. To avoid a systematic error, the order of light and dark measurements was switched from each measurement day to the other. Differences in N_2_O fluxes between treatments or diurnal and nocturnal periods (Day‐Night test) were calculated using the Wilcoxon test for paired samples (paired = T, alternative = “greater”). Differences were regarded significant for *p* ≤ .05.

For the Day‐Night test, the relationship between N_2_O fluxes and WFPS was tested using linear regression (lm function from the *stats* package).

## 3 RESULTS AND DISCUSSION

Light conditions had a reducing effect on observed N_2_O emissions for both net ecosystem fluxes from ash as well as beech and respective average fluxes were 65% and 34% lower compared to observations made under dark conditions. In both cases, the reduction (Δ) over the 58 days of measurements was significantly different from zero (*p* < .01). For the bare soil treatment, Δ was positive (5%) but no significant differences were found between light and dark measurements (*p* = .72). The average N_2_O emissions observed throughout the individual experiments are shown in Table 2.

**TABLE 2:** Average N_2_O fluxes [µg/m^2^] under dark and PAR conditions observed during the three experiments. For the net ecosystem experiment, both the daily average and average fluxes over the 2‐month period are presented. The data presented in this study originate from a running experiment and cover a period of 58 days (35 days after the first fertilization with 25 kg KNO_3_/ha) and includes 14 measurements. For the 24‐hr measurements, average values for each hour of the day (HOD) are shown. For the plant experiment, the cumulative fluxes over the 10‐day period are presented (see also Figure 2). Δ stands for the N_2_O_PAR_‐N_2_O_dark_). Different letters represent significant differences between the treatments

Time	Dark	PAR	Δ	Dark		PAR	Δ		Dark	PAR	Δ
	*Bare soil*			*F. excelsior*					*F. sylvatica*		
Net ecosystem experiment											
Sampling	*Daily measurements*										
1	61 (65)	85 (56)	24 (26)	23 (19)		6 (20)	−17 (13)		29 (31)	−1 (33)	−30 (14)
2	241 (225)	237 (209)	−4 (23)	19 (17)		11 (11)	−8 (11)		99 (27)	68 (31)	−31 (7)
3	84 (80)	75 (66)	−9 20)	13 (9)		−9 (11)	−22 (13)		40 (23)	15 (31)	−25 (14)
4	253 (246)	272 (244)	19 (32)	19 (16)		0 (11)	−19 (8)		122 (94)	113 (128)	−10 (34)
5	43 (56)	38 (41)	−5 (18)	11 (7)		−18 (15)	−29 (19)		14 (8)	−17 (11)	−30 (4)
6	188 (213)	165 (200)	−23 (20)	27 (24)		−6 (16)	−34 (26)		36 (23)	6 (25)	−30 (15)
7	40 (23)	44 (39)	4 (27)	23 (33)		−2 (14)	−25 (20)		27 (27)	3 (25)	−23 (17)
8	184 (186)	170 (173)	−14 (51)	154 (279)		112 (236)	−43 (43)		98 (80)	68 (76)	−30 (25)
9	69 (65)	65 (50)	−4 (17)	12 (13)		−14 (14)	−26 (13)		32 (24)	10 (21)	−22 (16)
10	278 (256)	294 (269)	16 (28)	102 (71)		51 (53)	−52 (22)		164 (142)	142 (136)	−22 (12)
11	66 (56)	67 (53)	1 (18)	17 (9)		−23 (8)	−40 (12)		69 (69)	39 (59)	−29 (32)
12	200 (169)	200 (173)	1 (21)	109 (108)		71 (87)	−38 (25)		323 (358)	248 (263)	−74 (118)
13	95 (144)	43 (44)	−52 (112)	84 (139)		37 (101)	−47 (179)		39 (40)	20 (42)	−19 (14)
14	72 (78)	203 (285)	131 (275)	33 (23)		14 (41)	−19 (47)		98 (82)	68 (104)	−30 (36)
*Average fluxes*											
	134 (162)	140 (172)	6 (85)	46 (94)		16 (78)	−30 (50)		85 (129)	56 (112)	−29 (37)
*cumulative fluxes*											
	1873 (1689)	1958 (1547)	84 (340)		646 (368)	228 (253)	−418 (280)	1,188 (793)		782 (756)	−406 (184)
HOD	24‐hr measurements										
5 a.m.				29 (13)		13 (17)	−15 (8)				
8 a.m.				45 (15)		23 (17)	−30 (4)				
11 a.m.				37 (25)		15 (20)	−29 (13)				
2 p.m.				40 (24)		18 (23)	−25 (6)				
5 p.m.				50 (31)		20 (24)	−23 (13)				
8 p.m.				44 (29)		36 (27)	−28 (9)				
11 p.m.				60 (38)		36 (37)	−23 (8)				
2 a.m.				48 (42)		31 (38)	−17 (9)				
Plant flux experiment											
*F. excelsior*											
				*Aboveground*					*Rhizosphere*		
				1,494 (1517)		508 (665)	−986 (852)		−50 (408)	−100 (355)	−51 (91)

**FIGURE 2:**
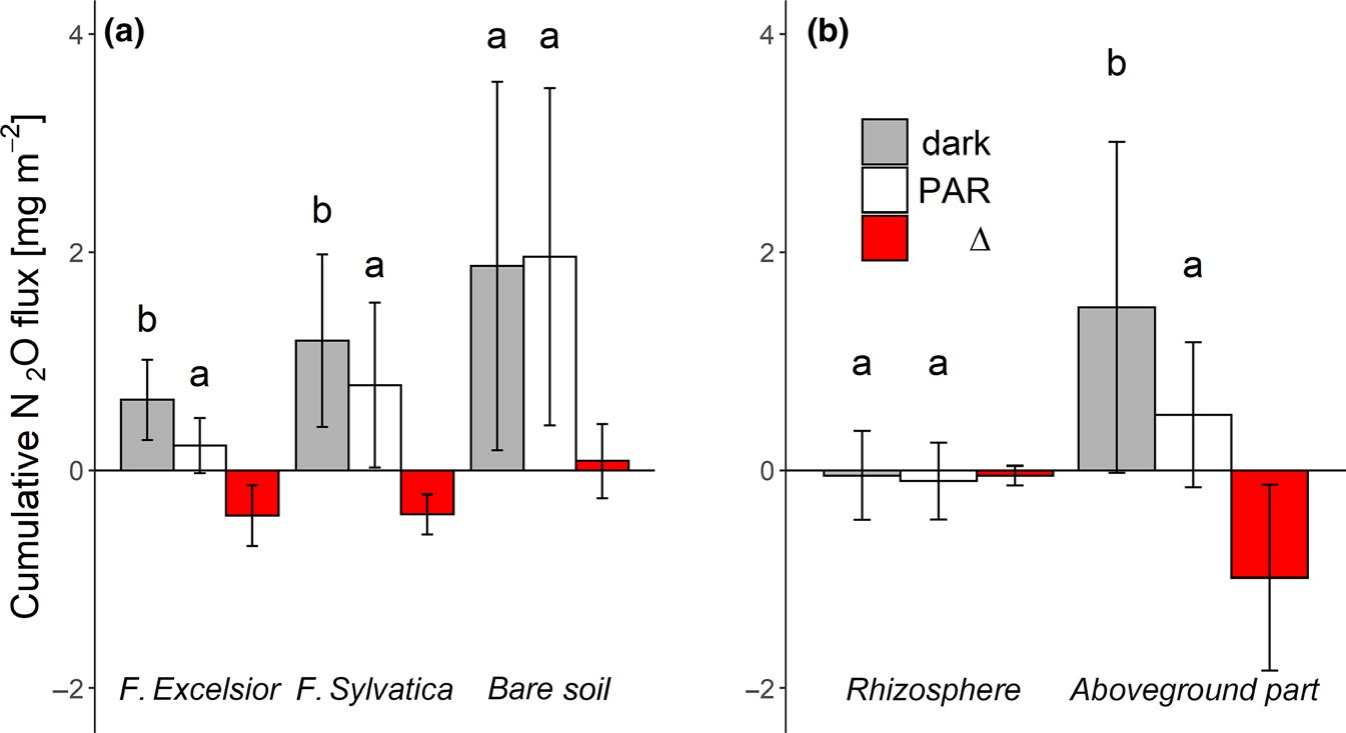
Mean net cumulative N_2_O fluxes under dark and light conditions and N_2_O reduction (Δ) from (a) ash, beech, and bare soil after the measuring period of 58 days and (b) from rhizosphere and aboveground biomass of ash after 4 days of measurements. Bars represent the standard deviation (*SD*). Different letters indicate significant differences (*p* < .05) between dark and PAR conditions. Differences between tree species (*Fraxinus excelsior*, *Fagus sylvatica*, and bare soil) and plant parts (rhizosphere and aboveground parts), respectively, were not significant (*p* > .05)

The metabolism of these tree species causes a strong reduction of net ecosystem N_2_O fluxes. Most likely, the N‐metabolism of the plant is involved, but C shortage by reduced rhizo‐deposition and water changes in the rhizosphere may also influence N_2_O emission from the soil. Considering only the rhizosphere of ash, as was done in the plant flux experiment, observed total N_2_O emissions were negative and this uptake was higher under PAR compared to dark conditions (Δ = 104%, *p* = .17; Figure 2b). A strong reduction of N_2_O fluxes by PAR was found from the shoots (Δ = −66%; *p* = .05), indicating that the photosynthetic effect mainly applies to the aboveground plant parts.

Generally, the cumulative N_2_O efflux from planted soil was lower than from bare soil and showed species‐specific differences confirming previous findings (e.g., Fender, Leuschner, Schützenmeister, Gansert, & Jungkunst, 2013; Fender et al., 2012). However, it mattered whether measurements were performed in darkness or under light. Ash caused a pronounced reduction of 66% in the dark and even 88% during the photosynthetic period in the light compared to bare soil. For beech, the corresponding decrease was less pronounced but still 37% in the dark and 60% in the light (Figure 2a). If photosynthetic N‐assimilation alone were responsible for the decrease in N_2_O efflux, this effect should cease in the dark, reaching similar rates as the bare soil. However, in the dark, both tree species revealed highly relevant reductions of N_2_O emissions compared to the bare soil, whereas ash showed a stronger effect than beech.

**FIGURE 3:**
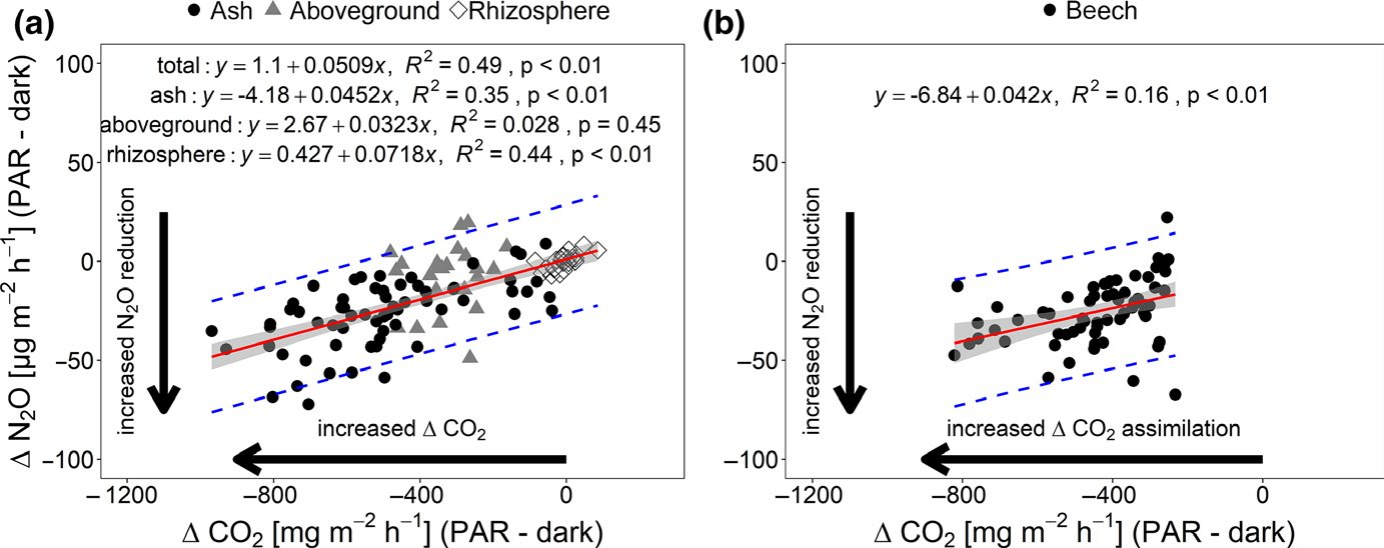
Relationship between plant CO_2_ assimilation (Δ CO_2_) and N_2_O reduction (Δ N_2_O) for ash (a) and beech (b). The linear regression line (red), the 95% confidence interval (gray‐shaded area), and the prediction interval (dashed blue lines) are shown. N_ash_ = 64, N_aboveground_ = 23, N_tot_ = 87, N_beech_ = 64

Since ${\text{NO}}_{3}^{-}$ uptake by plant roots was found to start with a lag time of 4 hr after transition from dark to light (Delhon, Gojon, Tillard, & Passama, 1996), it appears unlikely that N uptake by plants has an immediate effect on N_2_O emission from the soil. The short‐term experiment rather shows that aboveground plant parts may have a direct effect on N_2_O emissions. Both ash and beech with soil in the net ecosystem experiment showed a strong negative correlation between plant CO_2_ assimilation and decrease in N_2_O efflux (Figure 3a,b). However, a closer look at the aboveground plant part during the plant experiment revealed no such correlation (Figure 3a).

**FIGURE 4:**
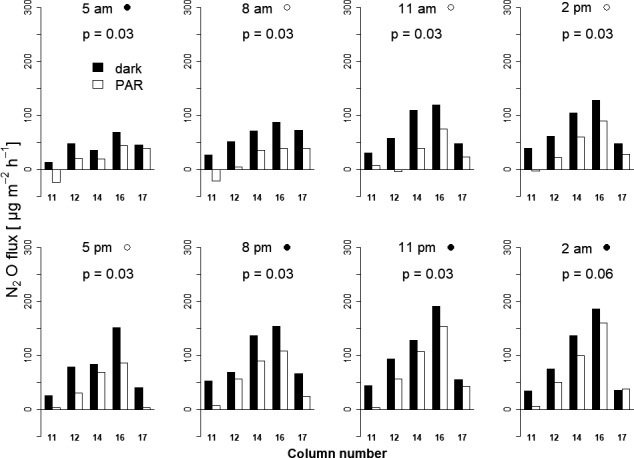
N_2_O fluxes (µg m^2^ hr) of each ash planted column under dark and PAR conditions. ●, nighttime, ○, daytime

Photosynthesis has an instantaneous and species‐specific effect on the reduction of N_2_O emissions from the soil. To prove whether this effect might be a function of the trees’ photoperiodism, we measured N_2_O efflux rates of eight dark–light intervals throughout a diurnal course over 24 hr. The amount of the photosynthetically induced reduction of N_2_O efflux was influenced by the time of the day (Figure 4), and Δ was higher during the diurnal (−56%) compared to the nocturnal (−34%) period (*p* = .01). The reducing effect of photosynthesis was apparent throughout the day and Δ was significantly different from zero (*p* = .03) for all but the last measurements (*p* = .06; Table 2; Figure 4). An observed overall trend of increasing N_2_O efflux can be related to the decreasing water content in the soil columns during the 24‐hr experimental period (*R* ^2^ = 0.84; *p* < .01 and .93; *p* < .01, in the dark and in the light, respectively), which most likely explains the diurnal effect best.

This study provides evidence that tree photosynthesis can have a considerable and instantaneous reducing effect on N_2_O emission from ecosystems. This effect was highly repeatable in our experiments, and tree species‐specific differences were found. The effect was persistent independent of the order of dark before light or dark after light measurement. The plant‐induced reduction of N_2_O efflux under light may hold key information in understanding the underlying processes driven by N assimilation of tree roots during darkness. Our results showed that the photosynthetic effect does not apply to the rhizosphere but primarily to the aboveground plant parts. However, the correlation between N_2_O reduction and CO_2_ assimilation (Figure 3) of the net ecosystem experiment suggests that there might be an effect on the rhizosphere that has not come to light in the experiments presented here. In contrast to leaves, root‐specific plasma membrane‐bound nitrate reductase (PM‐NR) is not down‐regulated in the dark so that apoplastic reduction of ${\text{NO}}_{3}^{-}$ to ${\text{NO}}_{2}^{-}$ can take place at similar rates day and night (Duncanson, Ip, Sherman, Kirk, & Wray, 1992; Eick & Stöhr, 2012; Stöhr & Mäck, 2012). In the apoplast, toxic levels of nitrite are avoided by further metabolization to NO via a plasma membrane‐bound nitrite/NO reductase (NI‐NOR), which is spatially associated to PM‐NR (Delhon et al., 1996). Whether the symplastic pathway of ${\text{NO}}_{3}^{-}$ reduction to ${\text{NH}}_{4}^{-}$ in the roots also contributes to ${\text{NO}}_{3}^{-}$ uptake in the dark requires further investigations. Nevertheless, the ATP and reductant that are required for the N assimilation during which N_2_O is being produced may be provided by photosynthesis, respiration or both—in any case does N assimilation depend on increased respiratory carbon flow (Turpin, Weger, & Huppe, 1997). As summarized by Huppe and Turpin (1994), in the presence of an alternative carbon source, the inhibition of ${\text{NO}}_{3}^{-}$ assimilation can be overcome. This has been demonstrated by darkened photosynthetic tissues of higher plants, showing that N_2_O production during N assimilation also occurs in the absence of plant photosynthesis. This finding has been confirmed by Zou et al. (2005) who found a correlation between N_2_O emissions from plants and a plant respiratory coefficient, indicating that N_2_O emissions from plants might be associated with plant respiration. Maintaining a high but physiologically tolerable level of apoplastic NO_2_‐ in the dark appears to provide the physiological prerequisite for the correlation between increasing photosynthesis and decreasing denitrification in the light because nitrite reductase (NIR) in the chloroplast is not substrate limited, particularly not after the onset of light. Moreover in relation to the soil, plant‐mediated denitrification (Delhon et al., 1996) in the dark is of minor significance, if at all, because otherwise a decrease in N_2_O efflux would not occur. The coupling of N metabolizing processes in the dark and in the light is ecologically meaningful, because in nature, ash is distinguished by a strong growth under light‐limited conditions. The fact that beech also caused a considerable reduction of N_2_O emission in the light, but with a weaker, albeit significant, correlation between photosynthesis and N_2_O decrease, gives rise to the assumption that, in the dark, ${\text{NO}}_{3}^{-}$ reduction in beech roots might be less pronounced than in ash. The lower reduction of N_2_O emission from beech‐planted columns in the dark supports this assumption.

We are aware of the fact that the observed N_2_O reduction potential under illuminated conditions of 65% by ash and 31% by beech does not allow for global projections yet, but we consider these results as an important step into further investigations on the disentanglement of N metabolizing pathways in plants and its coupling with N_2_O release from the soil. This will considerably improve our understanding of the temporal dynamics of N_2_O emissions. Studies on N_2_O emissions using eddy covariance technique are still scarce, especially for forest sites, but diurnal patterns of N_2_O emissions have been found from a poplar plantation (Zona et al., 2013). Our results moreover show that our assumption of a non‐relevant effect on net ecosystem fluxes does not apply. The importance of considering photosynthesis in N_2_O budgeting has been suggested before. According to Bruhn et al. (2014), ecosystem N_2_O emissions were 30% higher under natural UV radiation compared to darkness. Mueller (2003) stated that emissions could be even twice as high compared with dark chambers.

Investigations on N_2_O fluxes from shoots of adult trees are difficult and measurements in the laboratory are mainly performed using saplings, while field measurements focus on the stems (Barba et al., 2019; Díaz‐Pinés et al., 2016; Machacova et al., 2013, 2016; Wen et al., 2017). Even though the importance of the latter as a N_2_O source is not clear and stems were found to be both a source (Díaz‐Pinés et al., 2016; Machacova et al., 2013, 2016; Wen et al., 2017) and a sink (Barba et al., 2019), excluding the contribution of trees to N_2_O exchange with the atmosphere might result in a systematic underestimation of the total forest ecosystem fluxes (Machacova et al., 2016).

Certainly, the influence of plants on the exchange of N_2_O between terrestrial ecosystems and the atmosphere is not limited to the competition with soil microorganisms for N without species‐specific differences. Obviously, the interaction between root‐mediated N‐metabolism and photosynthetic N‐assimilation of plants is still poorly understood and our knowledge on terrestrial ecosystem–atmosphere exchanges of N_2_O is to be expanded. Our results proved us partly wrong and have shown that we cannot predict ecosystem net fluxes by measuring plant‐derived and soil‐derived N_2_O fluxes separate from each other.

## CONFLICTS OF INTEREST

The authors declare no conflict of interest.

## AUTHORS' CONTRIBUTIONS

K.S. and H.F.J. conceptualized the lab studies; K.S., H.F.J., and D.G. designed the lab studies; K.S., M.G., K.H.E.M., and A.B.D.H. performed the studies, analyzed the samples and data; K.S., K.H.E.M., M.G., D.G., A.B.D.H., and H.F.J. wrote the paper. All authors discussed the results and commented on the manuscript.
